# Cueing natural event boundaries improves memory in people with post-traumatic stress disorder

**DOI:** 10.1186/s41235-023-00478-x

**Published:** 2023-04-27

**Authors:** Barbara L. Pitts, Michelle L. Eisenberg, Heather R. Bailey, Jeffrey M. Zacks

**Affiliations:** 1 Kansas State University, Manhattan, KS 66506 USA; 2 Washington University in St. Louis, St. Louis, MO 63130 USA

**Keywords:** PTSD, Symptom severity, Event segmentation, Memory, Cueing

## Abstract

People with post-traumatic stress disorder (PTSD) often report difficulty remembering information in their everyday lives. Recent findings suggest that such difficulties may be due to PTSD-related deficits in parsing ongoing activity into discrete events, a process called *event segmentation*. Here, we investigated the causal relationship between event segmentation and memory by cueing event boundaries and evaluating its effect on subsequent memory in people with PTSD. People with PTSD (*n* = 38) and trauma-matched controls (*n* = 36) watched and remembered videos of everyday activities that were either unedited, contained visual and auditory cues at event boundaries, or contained visual and auditory cues at event middles. PTSD symptom severity varied substantial within both the group with a PTSD diagnosis and the control group. Memory performance did not differ significantly between groups, but people with high symptoms of PTSD remembered fewer details from the videos than those with lower symptoms of PTSD. Both those with PTSD and controls remembered more information from the videos in the event boundary cue condition than the middle cue or unedited conditions. This finding has important implications for translational work focusing on addressing everyday memory complaints in people with PTSD.

## Introduction

Approximately 5% of US adults experience clinical levels of post-traumatic stress disorder (PTSD) following a life-threatening event (Perrin et al., 2014). Individuals with PTSD experience intrusive reminders of the traumatic event, changes in cognition and mood, general hyperarousal, and they actively avoid anything associated with the event. Many of the symptoms of PTSD involve memory problems for the traumatic event, such as vivid flashbacks, life-like nightmares, intense negative feelings about the event, and detachment from event reminders long after the event has ended (American Psychiatric Association, 2013). In fact, many current theories of PTSD propose that memory abnormalities are central to the development and persistence of symptoms (Beierl et al., 2020; Brewin, 2018; Rubin et al., 2008). For example, Brewin (2011, 2014) proposed that PTSD stems from incomplete long-term memory representations that don’t accurately reflect sensory input. Some theories of PTSD further propose an attentional bias toward perceptual details of an event over conceptual information (Ehlers & Clark, 2000). According to this perspective, high basal arousal levels induce data-driven processing of ongoing activity, which results in memory representations of events that have rich perceptual information and poor event structure (i.e., disorganized and incoherent), with minimal contextual information. These characteristics make such memories difficult for people with PTSD to voluntarily search for and retrieve information from episodic memory (Sherrill & Magliano, 2017). This is consistent with findings from Sherrill and Magliano (2017) that state anxiety increases perceptual processing over conceptual processing. These processing deficits are proposed to not just be a side effect of PTSD symptoms, but Ehlers and Clark (2000) also proposed that they contribute to and maintain the disorder. Therefore, understanding these underlying memory deficits may be crucial for developing treatments and improving functional outcomes (Scott et al., 2015).

In addition to trauma-related memory disturbances, people with PTSD often report trouble remembering aspects of everyday life. For example, combat veterans with PTSD report higher frequency of forgetting everyday things, such as names and appointments and decreased use of mnemonics, than combat veterans without PTSD (Carlozzi et al., 2011). These real-world memory failures are consistent with previously reported PTSD-related differences in memory using neuropsychological measures. These studies find that verbal memory, in particular, is significantly worse in patients with PTSD than those without (Johnsen & Asbjornsen, 2008). These PTSD-related memory deficits are associated with poor social and occupational functioning (Geuze et al., 2009) and worse treatment outcomes (Wild & Gur, 2008).

Despite subjective memory complaints and objective neuropsychological deficits associated with PTSD, previous experimental studies often fail to find differences in real-world memory (Carlozzi et al., 2011; Roca & Freeman, 2001). This discrepancy between memory findings may be due to the use of simple verbal or visual materials in standardized objective measures, which may not reflect real-world memory difficulties. However, more recently, we have demonstrated objective PTSD-related memory deficits using real-world video stimuli: People with higher PTSD severity recalled fewer fine-grained actions from videos of everyday events than did control subjects who also had a history of trauma (Pitts et al., 2022) and more severe PTSD symptoms were related to worse memory performance (Eisenberg et al., 2016; Pitts et al., 2022). In addition to these objective memory deficits, we also found that participants with higher PTSD symptom severity were less able to effectively encode the to-be-remembered activity, suggesting a potential mechanism to explain memory deficits associated with PTSD.

According to Event Segmentation Theory (Zacks et al., 2007), effectively encoding ongoing activity requires the perceptual system to break up or “chunk” activity into discrete events. Incoming sensory information along with knowledge from relevant previous experiences is mentally represented in an event model in working memory. This event model guides comprehension of ongoing activity and contributes to forming predictions about what will happen next. Event Segmentation Theory proposes that when a new phase of activity begins, activity becomes less predictable, and prediction error spikes. In response, the cognitive system updates its current event model. In most cases, prediction error falls after this updating and comprehension goes on smoothy. The point in time at which an event model is updated is perceived as an *event boundary*.

In the laboratory, researchers can measure the ability to identify event boundaries using the unitization task (Newtson, 1973), in which viewers are asked to press a button whenever they judge that one meaningful activity ends and another begins. Importantly, viewers tend to largely agree on where they perceive event boundaries using this task (Speer et al., 2003; Zacks, Speer, Vettel, & Jacoby, 2006). However, deficits in event perception are implicated in several mental health conditions, including PTSD (Eisenberg et al., 2016), schizophrenia (Zalla et al., 2004), obsessive compulsive disorder, and Parkinson’s disease (Zacks & Sargent, 2010). For example, individuals with PTSD have been found to identify more idiosyncratic event boundaries compared to control subjects, suggesting a deficit in how they process and encode ongoing activity (Pitts et al., 2022). This deficit in event segmentation has implications for memory, as segmentation ability predicts subsequent memory over and above other cognitive abilities (Flores et al., 2017; Sargent et al., 2013). Consistent with this relationship, segmentation ability mediates the relationship between PTSD and memory performance, such that PTSD is associated with lower segmentation agreement, which in turn is associated with lower memory performance (Pitts et al., 2022).

If people who are better at identifying event boundaries have better memory for events, does drawing attention to event boundaries improve memory? Gold et al. (2017) investigated this question in older adults, who show deficits in both event segmentation and episodic memory (Pitts et al., 2021; Sargent et al., 2013; Zacks et al., 2006). They cued viewers to when normative event boundaries occurred in movies by slowing and pausing movie playback at boundaries, playing a tone, and using an arrow to indicate the object currently being acted on. Both older and young viewers’ memories benefited from this encoding manipulation. Therefore, the results of Gold et al. support the hypothesis that intervening to support segmentation can improve memory in people with memory deficits.

The cueing paradigm used by Gold et al. (2017) affords us the opportunity to evaluate whether segmentation ability plays a causal role in memory deficits associated with PTSD. If improving event segmentation via cueing also improves memory, then event segmentation could inform interventions for PTSD. Previous empirical and theoretical results would not lead one to propose that improving event encoding would affect processing of traumatic memories, but it could improve comprehension and memory of everyday events, potentially leading to improved cognitive performance and subjective sense of functioning.

In the current study, people with PTSD and controls watched and remembered videos of everyday activities that were either unedited, contained visual and auditory cues at event boundaries, or contained visual and auditory cues at event middles. We predicted that people with PTSD would show worse memory compared to controls for events in the unedited condition and that cueing event boundaries would improve memory for both groups and could potentially reduce or even eliminate PTSD-related memory deficits. Recent approaches to characterizing psychopathology, including PTSD, suggest that psychopathology is best measured on a continuous, rather than a dichotomous, scale. Therefore, we included a continuous measure of PTSD severity. We predicted that people with higher PTSD severity would show worse memory for events in the unedited condition and that cueing event boundaries would improve memory across the severity range, with a potentially greater impact at the higher severity ranges.

## Method

### Participants

Participants were 18- to 50-year-olds with a history of a traumatic life experience who had already participated in another study in the same laboratory (Pitts et al., 2022). All participants were originally recruited from the Volunteer for Health participant registry, a subject pool maintained by the Washington University School of Medicine, and from advertisements posted on Saint Louis Craigslist. All participants in that study were diagnosed using the Structured Clinical Interview for DSM-IV-Research Version (SCID-IV; Mood, Substance Use, PTSD, and Psychosis Modules; First et al., 2002) to determine assignment to PTSD or control groups. Control participants were recruited to match PTSD participants on age (within 10 years), gender, years of education (within approximately two years), and ethnicity (if mixed ethnicity, at least one match) and were required to have experienced a traumatic event that met the A1 DSM-IV criterion for PTSD.

Exclusion criteria for the PTSD group included no PTSD diagnosis, history of psychosis, current substance use disorder, and current manic episode. Exclusion criteria for the control group included more than three current PTSD symptoms, PTSD symptoms that significantly interfered with important life functioning or that caused significant distress, history of psychosis, current substance use disorder, and current manic episode. A total of 38 people with PTSD and 36 controls who completed the first study also agreed to participate in the current study. Table 1 provides demographic information on the final sample included in the current study.

**Table 1:** Demographic and diagnostic characteristics of sample

	PTSD	Control	*p*-value
Final sample	38	36	
Mean age in years (SD)	34.58 (8.69)	34.25 (8.73)	0.871
Mean years of education (SD)	13.92 (1.98)	14.81 (1.70)	0.044
*Gender*			
Male	8	6	
Female	30	30	
*Racial identification*			
White	22	27	
Black	9	8	
Asian	2	0	
Mixed race	4	1	
Unknown	1	0	
*Mean (SD) psychological scores*			
PCL	55.53 (13.69)	20.39 (6.04)	< .001
DASS	61.84 (24.46)	9.22 (15.87)	< .001
DES	634.87 (448.13)	204.72 (242.34)	< .001
LSAS	69.12 (30.28)	30.50 (20.57)	< .001
MPSS	48.84 (19.07)	64.03 (17.05)	.001

PCL, PTSD Checklist; DASS, Depression Anxiety Stress Scale; DES, Dissociative Experiences Scale; LSAS, Liebowitz Social Anxiety Scale; MPSS, Multidimensional Scale of Social Support

Participants in both the PTSD and control groups reported experiencing, witnessing, and learning about a wide range of traumatic events, as measured using the Life Events Checklist of the PTSD Checklist for DSM-IV—Civilian Version (PCL-C; Weathers et al., 1993). These traumatic experiences included transportation accidents (5 in the PTSD group, 15 in the control group), physical assault (12 in the PTSD group, 5 in the control group), sexual assault or unwanted sexual experiences (14 in the PTSD group, 6 in the control group), combat exposure (2 in the PTSD group, 0 in the control group), life threatening illness/injury or sudden violent or accidental death (2 in the PTSD group, 8 in the control group), and other noncategorized events (0 in the PTSD group, 2 in the control group). Many participants reported experiencing multiple different types of traumatic events.

### Design

The experimental design was a 3 × 3 within-participants design in which participants watched movies that included cues that marked *event boundaries*, included cues that marked *event middles,* or included no cues (*unedited* movies). After watching the movies, participants recalled what happened in the movies. We scored each participant’s recall of each movie for information from event boundaries (which was cued in the event boundary condition), from event middles (which was cued in the event middle condition), and information that was not cued in any condition (uncued information).

## Materials and measures

### Videos

**Fig. 1:**
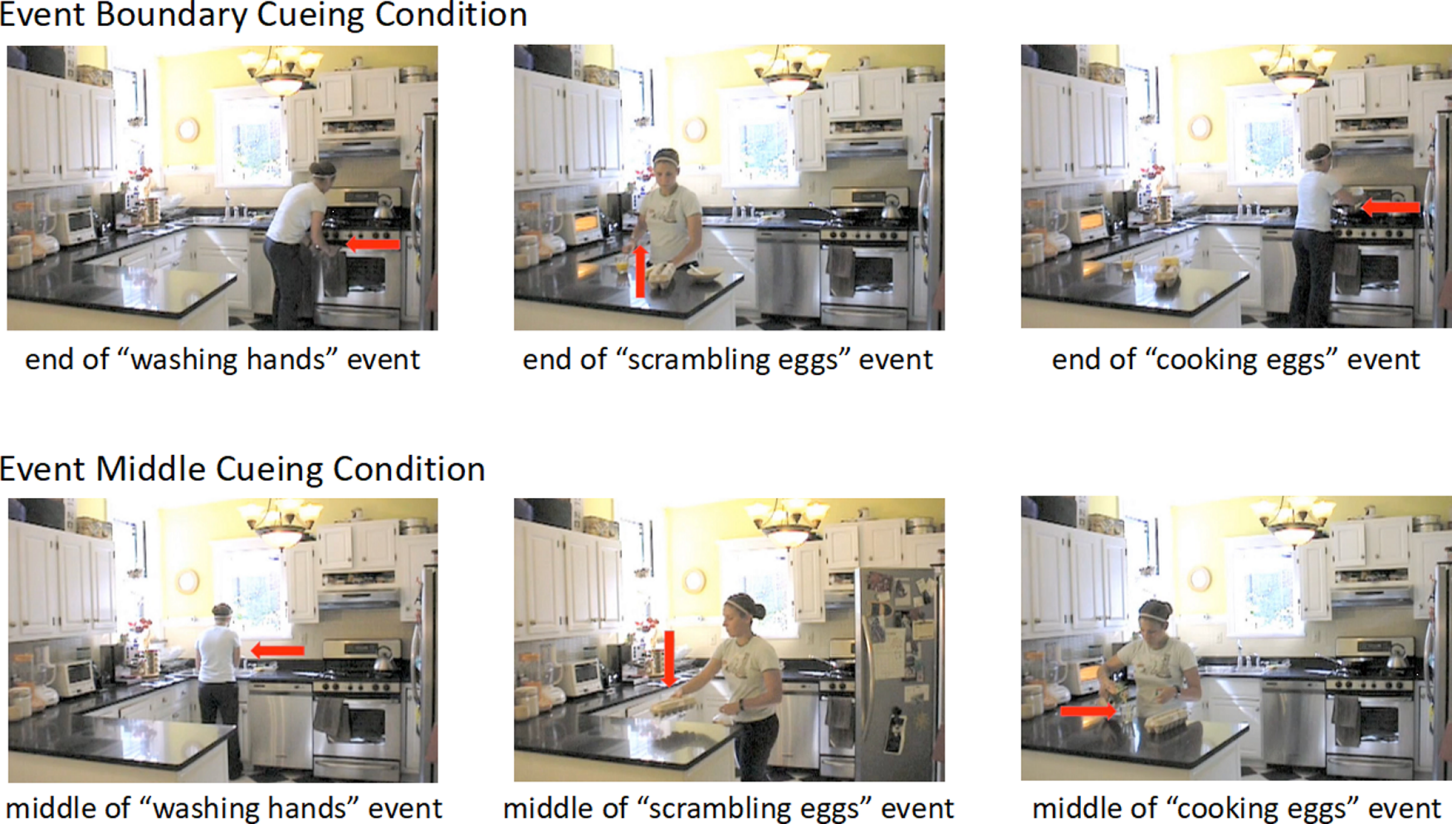
Examples from the event boundary (top) and event middle (bottom) cueing conditions. Participants experienced a brief slowing of the video, saw an arrow pointing toward the object being interacted with, and heard a bell sound either at an important change (event boundary cueing condition) or in the middle of an unfolding event (event middle cueing condition)

Three videos were shot at a rate of 25 fps and depicted actors (college students) performing activities typical in everyday life, including making breakfast (329 s), preparing for a party (376 s), and planting plants (354 s). All videos were filmed from a fixed, head-height perspective, with no pan or zoom. Three versions of each video were created to correspond to the following cueing conditions: (1) unedited; (2) event boundary: boundaries identified reliably in previous studies were cued by a brief slowing of the video, the presence of an arrow pointing toward the object being interacted with, and a bell sound; and (3) event middle: time points at the temporal middle of two boundary points were cued in the same way. Examples of the stimuli are shown in Fig. 1 and full stimuli are available at https://osf.io/ht4fg/.

### Psychological assessments

All psychological assessments were administered at the time that participants completed session one of the first study. Clinical diagnoses, including a diagnosis of PTSD, were assessed using the SCID-IV. The SCID is one of the most widely used diagnostic instruments in clinical research and has high clinical validity and reliability (First & Gibbon, 2004). The SCID-IV was modified to include criteria for both DSM-IV and DSM-5, as the SCID for DSM-5 had not yet been released at the time the study began.

The PTSD Checklist for DSM-IV—Civilian Version (PCL-C; Weathers et al., 1993) measured self-reported symptoms of PTSD identified by the DSM-IV-TR (American Psychiatric Association, 2000). Respondents rated on a 5-point scale ranging from 1 (*not at all*) to 5 (*extremely*) how much “you have been bothered by that problem in the past month.” The 17 items were summed for a total score with a possible range of 17–85. This brief screening tool is one of the most well-accepted tools for assessing PTSD symptoms. The updated version of this measure (PCL-5; Weathers et al., 2018) was not available when data collection first began in 2013.

Additionally, participants completed the Depression Anxiety Stress Scale-42 (DASS; Lovibond & Lovibond, 1995), the Dissociative Experience Scale (DES; Bernstein & Putnam, 1986), the Liebowitz Social Anxiety Scale (Liebowitz, 1987), and the Multidimensional Scale of Perceived Social Support (MPSS; Zimet et al., 1988). See Table 1 for scores on all these measures by group.

### Free recall task

Immediately after watching each video, participants were given seven minutes to type as much as they could remember from the video they had just watched, in the order they remembered the activity occurring. To score the free recall, research team members constructed a list of the basic actions performed by the actor in the video using the action coding system (ACS) described by Schwartz et al. (1991). After familiarization with the scoring procedure, each research team member scored ten participant responses for one video, and item scores were compared with those of one of the lead researchers. This initial scoring produced an interrater Kappa = 0.90. Discrepancies were reviewed and discussed to agree on general scoring principles. Each team member then coded the remaining participant recall responses for one video. Actions were categorized into three information types: (1) uncued information: actions that were not cued in any condition; (2) boundary information: actions that occurred at a boundary location and were thus cued in the event boundary condition; or (3) midpoint information: actions that occurred at a midpoint between boundaries and were thus cued in the event middle cueing condition. Accurate recall of each action (1 or 0) was the dependent measure. Of note, responses to this task generally do not contain inaccurate information or intrusions. We report mean probability of recall (based on predicted values from the models.)

### Procedure

Participants completed all tasks during one session that lasted approximately 1.5 h and that occurred shortly after their participation in last session of their previous study in the laboratory (Pitts et al., 2022). (During that study, participants watched movies of everyday activities similar to the ones used in this experiment, segmented those movies into meaningful events, and completed memory tests.) During this session, participants completed informed consent and watched three videos of everyday events. Each participant watched one unedited movie, one movie cued at event boundaries, and one movie cued at event middles. While the order of movies was not counterbalanced across participants (all participants watched the making breakfast movie, followed by the preparing for a party movie, and then the planting plants movie), the order of the edited conditions was counterbalanced across participants. For example, some participants watched the unedited breakfast movie, followed by the event boundary preparing for a party movie, and then the event middle planting plants movie, whereas other participants watched the event middle breakfast movie, followed by the unedited preparing for a party movie, and then the event boundary planting plants movie. All possible orders of the cuing condition were included in the counterbalancing. After watching each movie, participants completed the free recall task described above. Throughout movie viewing, participants right eyes were tracked with an EyeLink 1000 (SR Research Ltd, Mississauga, ON, Canada) to allow for potential future exploratory analyses of effects of the manipulation on visual focus; those analyses are not included here.

## Results

Here, we report analyses testing the prediction that people with PTSD would show worse memory compared to controls for events in the unedited condition and that cueing event boundaries would improve memory for both groups and could potentially reduce or even eliminate PTSD-related memory deficits. We tested two models: one to test the effect of the diagnosis of PTSD and a second to test the effect of PTSD symptom severity.

### Group analysis

To determine whether the cueing intervention affected recall memory, a binomial mixed-effects regression model examined the fixed effects of Group (PTSD vs. control), cueing condition (unedited, event boundary, event middles), and information type (boundary, midpoint, uncued), and their interaction on recall memory for each action item. The model also included random intercepts for Subject and Video. Model effects are shown in Table 2. Effect sizes are represented by R squared values for the full model (*R*^2^_*m*_ theoretical; Rights & Sterba, 2020).

**Table 2:** Model estimates for all independent variables

Variable	Wald	*df*	*p*
Group	2.62	1	.105
Cueing condition	13.66	2	.001
Information type	52.10	2	< .001
Group × condition	4.89	2	.086
Group × information type	0.17	2	.920
Condition × information type	5.80	4	.215
Group × condition × information type	2.70	4	.609

**Fig. 2:**
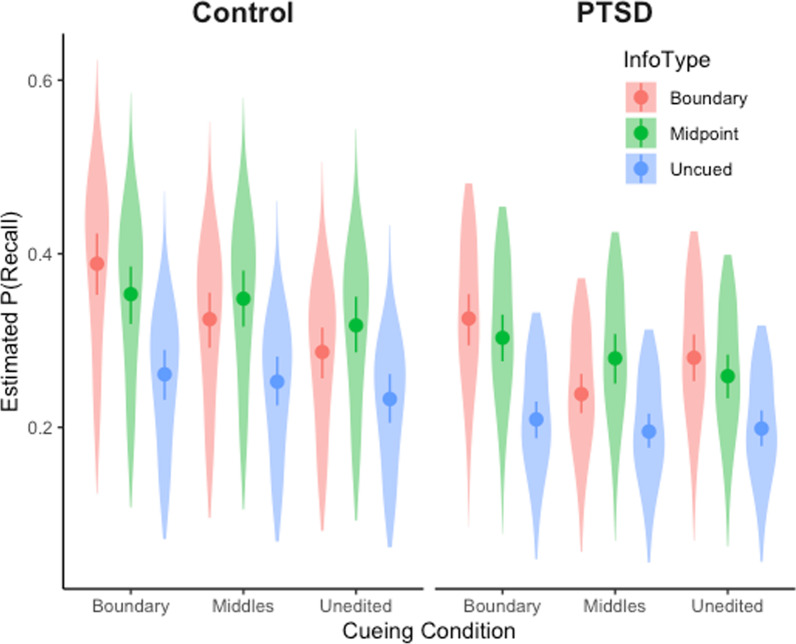
Recall probability as a function of group, cueing condition, and information type. Points represent means and whiskers show 95% confidence intervals

As expected, people with PTSD (*M* = 0.24, SE = 0.02) recalled less than controls (*M* = 0.29, SE = 0.02); however, this difference was not significant. As shown in Fig. 2, there was a significant effect of cueing condition, such that subjects were more likely to recall information from the event boundary cueing condition (*M* = 0.29, 95% CI 0.25, 0.33) than the event middles (*M* = 0.26, 95% CI 0.22, 0.30), *z* = 3.76, SE = 0.05, *p* < 0.001, and more likely to recall information from the event boundary cueing condition than the unedited condition (*M* = 0.25, 95% CI 0.22, 0.29), *z* = 4.86, SE = 0.06, *p* < 0.001. Recall did not differ between the unedited and event middle conditions, *z* = 1.11, SE = 0.05, *p* = 0.509. There was also a significant effect of information type, such that subjects were more likely to recall boundary information (*M* = 0.30, 95% CI 0.25, 0.34) than uncued information (*M* = 0.21, 95% CI 0.18, 0.25), *z* = 10.51, SE = 0.06, *p* < 0.001, and were more likely to recall midpoint information (*M* = 0.30, 95% CI 0.26, 0.34) than uncued information, *z* = 11.00, SE = 0.07, *p* < 0.001. Recall did not differ between midpoint information and boundary information, *z* = − 0.34, SE = 0.05, *p* = 0.939, suggesting that cueing improved memory for cued information.

None of the interactions were significant. The control group showed a slightly larger effect of the boundary cueing manipulation, but the interaction between group and cueing condition was not statistically significant. The full model explained 9.5% of the variance in memory performance.

### Symptom severity analysis

**Fig. 3:**
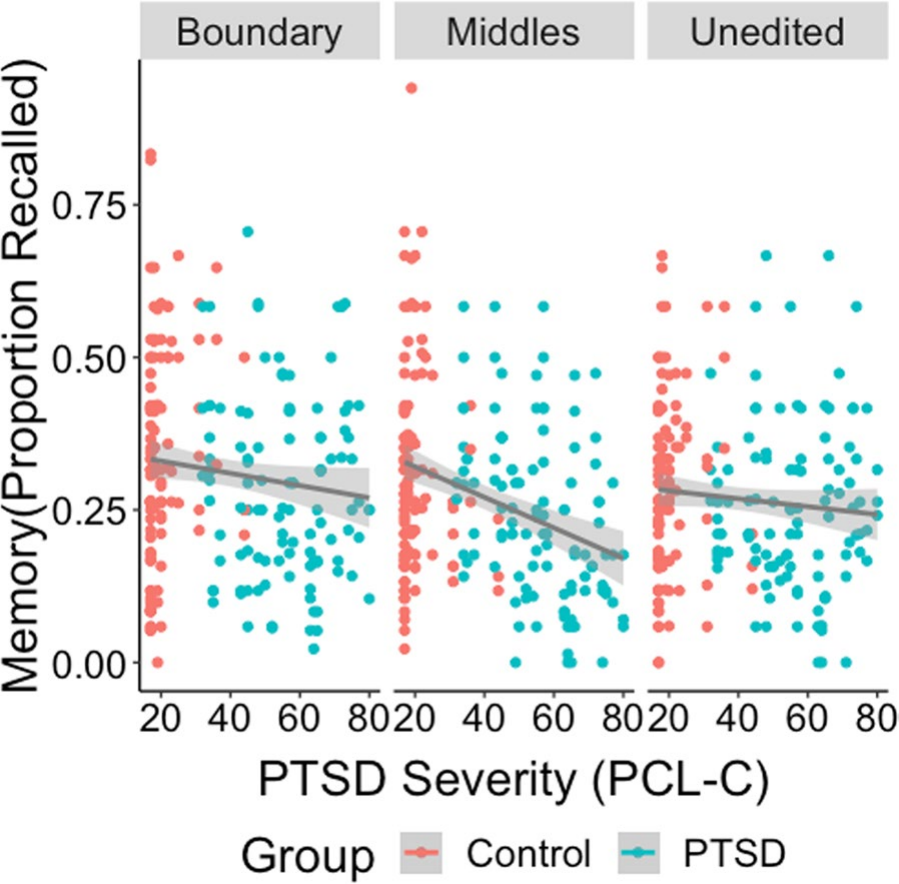
Recall probability as a function of PTSD symptom severity and cueing condition

We then examined the effects of PTSD symptom severity (PCL score), cueing condition (unedited, event boundary, event middles), and information type (boundary, midpoint, uncued), and their interaction on recall memory for each action item. The model also included random intercepts for Subject and Video. Model effects are shown in Table 3. As shown in Fig. 3, the effect of Symptom Severity was significant, such that higher symptom severity was associated with lower memory performance. The effects of cueing condition and information type were the same as those from the group analyses.

**Table 3:** Model estimates for all independent variables

Variable	Wald	*df*	*p*
PTSD symptom severity	6.68	1	.010
Cueing condition	22.64	2	< .001
Information type	200.14	2	< .001
PTSD symptom severity × condition	14.73	2	< .001
PTSD symptom severity × information type	1.22	2	.544
Condition × information type	10.51	4	.033
PTSD symptom severity × condition × information type	7.40	4	.116

There was also a significant interaction between symptom severity and cueing condition, such that there was a stronger negative effect of symptom severity on memory in the middle condition than in the boundary, estimate difference = 0.007, *z* = 2.99, *p* = 0.008, or unedited conditions, estimate difference = − 0.009, *z* = -3.85, *p* < 0.001 (Fig. 3). Lastly, the interaction between cueing condition and information type was significant, such that subjects were more likely to recall boundary information in the boundary condition than middle, *z* = 4.22, SE = 0.13, *p* < 0.001, or uncued information, *z* = 3.82, SE = 0.13, *p* < 0.001. None of the other interactions were significant. The full model explained 9.6% of the variance in memory performance.

## Discussion

The current study investigated whether cueing event boundaries would improve memory for everyday activities in people diagnosed with PTSD and matched controls. Participants from both groups were more likely to remember information from the videos in the event boundary cue condition than the middle cue or unedited conditions. In addition, people with higher PTSD severity remembered less information than people with lower PTSD severity. There was a significant interaction with cuing condition, such that people with higher severity of PTSD symptoms particularly struggled when event middles were cued. The main effect of PTSD severity on memory is consistent with results reported by Eisenberg et al. () and Pitts et al. (2022), who found that higher PTSD severity was associated with worse memory for everyday events. In addition, the form of the interaction suggests that while people with lower PTSD severity were able to compensate for being given unhelpful event cues and still perform well on recall, people with higher severity of PTSD symptoms particularly struggled with recall when given this unhelpful information. While this was not fully aligned with our initial prediction, it does suggest that unhelpful or incorrect structural cues may be particularly damaging for people with high PTSD severity. If future studies replicate this result, this finding could improve our understanding of the mechanisms involved in memory disruptions in people with severe PTSD.

On the other hand, there was not a significant difference in memory at the group (PTSD vs. control) level, though the results trended in this direction. Under current systems of diagnosis, psychopathology is diagnosed binarily (a person either has a diagnosis or does not); however, psychopathology exists on a continuum. Figure 3 clearly illustrates that this is the case in the current study: people who received a PTSD diagnosis had a wide range of severity scores on the PCL-C, overlapping substantially with the control group at the low to mid-severity levels. It is therefore unsurprising that the effect of PTSD on memory was stronger using the continuous measure compared with the dichotomous measure of group.

The finding that cues improved memory for people in both groups is consistent with Gold et al. (2017), who similarly found that cuing event boundaries improved memory for both older and young adults. Our findings extend these results to show improvements in memory for another population, people with PTSD, who exhibit deficits in both event segmentation and memory. These findings support the idea that segmenting ongoing activity into events provides structure for subsequent episodic memories (Ezzyat & Davachi, 2011; Gold et al., 2017; Zacks, 2020).

One possible interpretation of cue-related benefit in memory for event details is that the cueing procedure is increasing attention to the videos in general. This interpretation is consistent with the Attentional Boost Effect (Swallow & Jiang, 2013), which proposes that cues aimed to increase attention to one aspect of a task may also increase attention to other aspects and thereby boost overall performance. While increased attention to the videos likely increases overall performance, this general attention explanation cannot account for the finding that cueing event boundaries produces the largest benefit in memory. Rather, this finding supports the claim originally made by Gold et al. (2017) that the cueing procedure improves one’s ability to structure events.

Interestingly, both groups benefited from cueing event boundaries. The performance of those with PTSD increased with event boundary cueing to the level achieved by controls without cueing. However, cueing also improved memory in the controls, so the cueing intervention did not interact with the effect of group (see Fig. 2) and there was no significant difference in the slope of the relationship between PTSD and memory for the boundary cuing versus uncued conditions (see Fig. 3). A similar result was observed for age differences in previous work (Gold et al., 2017). One possibility is that the event segmentation intervention causally improved impaired memory encoding mechanisms in people with PTSD, as hypothesized, but also improved the efficacy of those mechanisms in control participants to an equivalent degree. An analogy may be helpful here: Suppose one had a go-kart that had developed a rusty axle. Greasing the axle would make it go faster by decreasing the friction caused by the rust. But greasing the axle of a non-rusty go-kart might also reduce friction and improve speed. An alternative possibility is that the cueing intervention boosts memory encoding in both groups by improving mechanisms that are not impaired by PTSD. An analogy here might be to improve the aerodynamics of the go-kart. This would make both the rusty and non-rusty go-karts go faster, by a mechanism unrelated to the impaired axle.

If this second possibility is the case, and the cueing manipulation does not affect a memory mechanism that is impaired in PTSD, what mechanisms might be responsible for PTSD-related deficits in everyday memory? One possibility is deficits that occur either earlier in the event processing stream, such as attention to the stimuli (Aupperle et al., 2012), or later, such as consolidation and retrieval. For example, previous research suggests that PTSD is associated with difficulty disengaging from one stimulus and redirecting attention to task-relevant stimuli (Pineles et al., 2009). Additionally, eye-tracking data from our own laboratory suggest that people with PTSD fail to accurately direct their gaze to where predictable actions are about to happen (Eisenberg et al., under review). Thus, there is some evidence of deficits in event processing that occur before encoding operations that are specific to forming an event model. A separate line of research suggests that drugs aimed at modulating traumatic memory during the early consolidation period may alleviate some of the fear and memory-related symptoms associated with PTSD (Parsons & Ressler, 2013). The early success of these treatments suggests that there may be PTSD-related deficits at the consolidation phase of event processing, but more research is needed to substantiate this as a general deficit in PTSD.

The fact that both groups benefited from cueing provides further evidence that event segmentation is causally related to memory encoding. This result converges with data from studies using controlled laboratory materials and more naturalistic stimuli (Clewett & Davachi, 2017; Pettijohn et al., 2016). It also suggests that the cueing paradigm is a promising avenue for improving memory for everyday activities within populations who have event processing deficits.

Helping people with PTSD to better understand event structure can improve their memory for events. In fact, Sherrill and Magliano (2017) suggested that established treatments for PTSD such as prolonged exposure therapy and cognitive processing therapy, which improve the organization of trauma memories, are likely leveraging event segmentation processes. Further research could evaluate ways to provide structural cues within past events, similar to what our paradigm does for current, ongoing events. For example, debriefing (Adler et al., 2009; Tuckey & Scott, 2014; but see Wesseley & Deahl, 2003) or guided journaling procedures (Ullrich & Lutgendorf, 2002) after a stressful experience may provide structure for memories that could enhance their representation in memory. Additionally, further research could evaluate the potential of using a training program, similar to the cueing procedure used here, to train people with PTSD to better attend to and understand event structure.

There are some limitations to our findings. First, PTSD diagnoses were based on a modified version of the SCID-IV, which may not elicit the same responses to those elicited by the updated version of this measure (SCID-5). Second, we did not investigate other cognitive abilities that may explain PTSD-related differences in memory; these could include attention, task set maintenance, memory consolidation, retrieval strategies, or knowledge about events and general information. Future research should investigate these as additional causal factors in the relationship between PTSD and objective memory problems. Third, the cueing procedure used here would need to be adapted to generalize to real-time naturalistic comprehension. Future research should investigate techniques for identifying event boundaries in real time and cueing them during real-world activity. Fourth, the participants in this study had previously participated in another study in which they segmented similar videos. It is possible, although unlikely, that their segmentation ability may have been improved by familiarization with the task. However, there is little reason to believe that experience with performing an event segmentation task would have such effects given that deliberate segmentation is stable over repeated viewings (Hard, Tversky, & Lang, 2006), even over a year’s delay (Speer et al., 2003). Fifth, all participants in this study previously participated in a study in which they performed an event segmentation task. While past experience with event segmentation has not been found to impact future performance on this task in healthy controls (Zacks et al., 2006), this has not been investigated in people with PTSD. This means that there is a small possibility that previous experience with this task impacted segmentation performance in participants with PTSD, leading to smaller group differences. Lastly, we did not collect data on other factors that may be important predictors of cognitive abilities and PTSD, such as socioeconomic status (DiGrande et al., 2008; Leonard et al., 2015).

In conclusion, this study found that a cognitive intervention to facilitate the encoding of event structure improved subsequent memory in people with and without PTSD. These findings provide further evidence that intervening to improve the initial encoding of event structure could be helpful in addressing everyday memory complaints in people with PTSD.

## Data Availability

The data and materials for all experiments reported in this manuscript are available at https://osf.io/ht4fg/. None of the experiments were preregistered.
